# Adverse skin reactions to personal protective equipment during COVID‐19 pandemic in Italian health care workers

**DOI:** 10.1111/dth.15460

**Published:** 2022-03-23

**Authors:** Ilaria Proietti, Ivan Borrelli, Nevena Skroza, Paolo Emilio Santoro, Maria Rosaria Gualano, Nicoletta Bernardini, Alessandra Mambrin, Ersilia Tolino, Anna Marchesiello, Federica Marraffa, Simone Michelini, Giovanni Rossi, Salvatore Volpe, Walter Ricciardi, Umberto Moscato, Concetta Potenza

**Affiliations:** ^1^ Department of Medico‐Surgical Sciences and Biotechnologies, Dermatology Unit “Daniele Innocenzi”, “A. Fiorini” Hospital Sapienza University of Rome Terracina Italy; ^2^ Department of Health Sciences and Public Health Università Cattolica del Sacro Cuore Rome Italy; ^3^ Department of Woman and Child Health and Public Health Fondazione Policlinico Universitario A. Gemelli IRCCS Rome Italy; ^4^ Department of Public Health Sciences and Paediatrics University of Torino Torino Italy

**Keywords:** COVID‐19, dermatology, personal protective equipment

## Abstract

To avoid exposure to SARS‐COV‐2, healthcare professionals must use personal protective equipment (PPE). Their use has been related to a series of adverse effects; the most frequent adverse events were headache, dyspnoea, and pressure injuries. Skin adverse effects are very common, including contact dermatitis, itching, erythema, and acneiform eruptions. The objective of this study is to evaluate the skin problems caused by personal protection equipment (PPE) in health care workers (HCWs) and to individuate eventual risk factors. From May to June 2020 a retrospective observational multi‐centric study conducted by an online survey sent by email, involving 10 hospital centers, was performed. We considered as independent variables gender and age, occupational group and sector, time of utilization, type and material of PPE. We tested 3 types of PPE: gloves, bonnet, and mask for different time of utilization (<1, 1–3, 3–6, >6 h). We performed a multiple logistic regression model to correlate them with skin adverse events occurrence. Among all the 1184 participants, 292 workers reported a dermatological pathology: 45 (15.41%) had psoriasis, 54 (18.49%) eczema, 38 (13.01%) acne, 48 (16.44%) seborrheic dermatitis, and 107 (36.64%) other. In our sample previous inflammatory dermatological conditions, female sex, prolonged use of PPE were significant risk factors for developing skin related adverse events considering all the PPE considered. The use of PPE is still mandatory in the hospital setting and skin adverse reactions still represent a global problem. Although data from Europe are limited, our study highlighted the importance of the problem of PPE skin reactions in a large sample of Italian healthcare professionals.

## 1 INTRODUCTION

Since December 2019, the severe acute respiratory syndrome coronavirus 2 (SARS‐CoV‐2) has rapidly spread worldwide causing more than 150 millions of cases and 3 millions of deaths, to date.

Considering the strong transmission of SARS‐CoV‐2 via virus‐containing droplets and contaminated objects, healthcare workers (HCWs) are considered a population at high risk of infection due the prolonged time of close contact with infected patients.

In Italy, according with the last data, 122,717 confirmed cases of infected health care workers were recorded, including 288 deaths (update February 24, 2021).^1^

As the pandemic increase, the use of personal protective equipment (PPE) has become essential for HCWs to fight safely against the virus.

In April 2020, World Health Organization (WHO) reported PPE as surgical masks, N95 or FFP2 masks, aprons, goggles, scrubs gloves, face shields, and alcohol‐based antiseptics and soaps are recommended to minimize the risk of infection.^2^

However, the extensive and prolonged use of PPE may cause various adverse skin reactions. For example, contact dermatitis caused by irritants and allergens contained in alcohol hand cleanser can occur in individuals with atopic predisposition. An excess of hand hygiene weaken the skin barrier and decrease the skin commensals.^3^

Moreover, a protracted use of masks and goggles is linked to contact dermatitis, itching, erythema and acneiform eruption mainly in nasal bridge, cheeks and chin. ^4^

The aim of this study was to evaluate the skin problems caused by personal protection equipment (PPE) in health care workers (HCWs) suggesting preventive measures to avoid the risk to develop skin diseases.

## 2 MATERIALS AND METHODS

This work was designed a retrospective observational multi‐centric study conducted by an online survey sent by email, involving 10 hospital centers (Milan, Rome, Latina, Catania, Naples) distributed throughout all the Italian territory carried out from May to June 2020. The data were obtained through a questionnaire was open to response in the period from November 2020 to January 2021. The survey was anonymous, and response to the questionnaire was voluntary. Dermatologists consented to participation in the research before filling the questionnaire. The questionnaire multiple‐choice questions covering demographic and professional data, previously diagnosed dermatologic diseases (by dermatological visit), new onset lesions and/or symptoms and their relationship with PPE use. Skin reactions on the body in HCWs who wore overalls have not been investigated.

We performed descriptive statistics to describe socio‐demographic aspects of participants and study variables characteristics, which were presented through theoretical score ranges, arithmetic means, standard deviations. Pearson bivariate correlations were performed to check multi‐collinearity and to give some preliminary information into relationships between dermatological disease and the use of PPE. *P* values were considered significant if they were <0.05.

In a second stage the significant predictors of the first stage were entered together in a final multiple logistic regression models. Data were stratified by gender, age, occupational group and sector, time of PPE utilization, type and material of PPE. Crude odds ratios (ORs) and adjusted ORs for all the other entered variables, along with 95% confidence intervals, were calculated. To analyze the collected data, we used the STATA 16 statistical package.

## 3 RESULTS

The sample included 1184 participants; skewness and kurtosis were used to investigate the distribution of the collected data and Shapiro‐Francia test was used to investigate normal distribution. Variables were not‐normally distributed, except for age, working sector, type of mask, adverse effects on hands, type of bonnet, number of work day loss and number of surveillance requests.

The sample was composed of 257 (21.71%) male and 927 (78.29%) female; the mean age was 43.37 (*SD* 10.94, range 21–68). As regards to occupational groups, the health care workers were distributed as follows: 332 (28.04%) physician, 772 (65.20%) nurse‐midwife, 17 (1.44%) nursing assistant, 26 ST (2.2%), and 37 others (3.12%). Concerning the working sector, 367 (31%) workers were employed in Hospital Ward (HW), 253 (21.37%) in ambulatory surgery, day hospital, ambulatory care unit workers, 114 (9.63) in Intensive Care Unit, 88 in (7.43) Emergency Room 71 (6%) in Surgery, and 250 (21.11) in other sectors.

Among all the participants, 292 workers reported a dermatological pathology nested in four different pathological groups: 45 (15.41%) had psoriasis, 54 (18.49%) eczema, 38 (13.01%) acne, 48 (16.44%), seborrheic dermatitis (SD) and 107 (36.64%) other.

We observed in 25 (2.11%) workers a loss of occupational days due to dermatological illness; in 56 times workers asked for occupational physician surveillance; in 30 cases, HCWs were removed from their workplaces and treated with specialist dermatological prescriptions.

We evaluated 3 types of PPE: gloves (Table 1), bonnet (Table 2) and mask (Table 3) for different hours of utilization per day (<1, 1–3, 3–6, >6) (Table 4).

**TABLE 1:** Number of health care workers (HCW) using different type of gloves and prevalence of dermatological disease

Type of gloves	Nr HCW	Prevalence (%)	Nr HCW with a Dermatological Pathology	Prevalence of Dermatological Pathology (%)
Latex no dust	385	32.54	82	28.08
Latex dust	83	7.02	14	4.79
Nitrile	671	56.72	181	61.99
Other	44	3.72	15	5.14

**TABLE 2:** Number of health care workers (HCW) using different type of mask and prevalence of dermatological disease

Type of mask	Nr HCW	Prevalence (%)	Nr HCW with a dermatological pathology	Prevalence of dermatological pathology (%)
Surgical mask	752	63.57	188	64.38
FFP2 no valve	375	31.70	96	32.89
FFP2 valve	15	1.27	1	0.34
FFP3 no valve	21	1.78	1	0.34
FFP3 valve	10	0.85	2	0.68
Other	10	0.85	4	1.37

**TABLE 3:** Number of health care workers (HCW) using different type of bonnet and prevalence of dermatological disease

Bonnet	Nr HCW	Prevalence (%)	Nr HCW with a dermatological pathology	Prevalence of dermatological pathology (%)
Cotton	249	37.84	65	35.91
NWF	349	53.04	99	54.70
Plastic	23	3.49	8	4.42
Tyvek®	24	3.65	5	2.76
Other	13	1.98	4	2.21

Abbreviation: NWF, non‐woven fabric.

**TABLE 4:** Stratified distribution of PPF per time of use

Time (hours)	<1 (all)	<1 (DP)	1–3 (all)	1–3 (DP)	3–6 (all)	3–6 (DP)	>6 (all)	>6 (DP)
Gloves	151 12.76	19 6.51	279 23.58	73 25.00	462 39.05	123 42.12	291 24.60	77 26.37
Mask	14 1.18	1 0.34	28 2.37	8 2.74	237 20.05	52 17.81	903 76.40	231 79.11
Bonnet	67 9.97	15 8.24	101 25.00	27 23.08	139 45.68	36 42.86	365 54.32	104 57.14

*Note*: all: entire sample; DP: health care workers with dermatological disease.

A total of 591 (49.92%) subjects reported adverse effects associated with the use of gloves; among them 203 presented a dermatological pathology (atopy or hand eczema, acne or seborrheic dermatitis). In 633 (53.46%) workers were detected mask‐related adverse effects; 197 of them presented a dermatological pathology. Finally we found 143 (15.84%) adverse effects associated with the use of bonnet; among them 58 presented a dermatological pathology.

We first analyzed the adverse effects on the hands; we found a statistically significant correlation (*p <* 0.05) between adverse effects and dermatological pathologies (atopic dermatitis [AD], SD, acne and psoriasis), age, sex, and time of utilization (Table 5). There was no correlation with the type of gloves (*p =* 0.28).

**TABLE 5:** Results from multiple logistic regression analysis with adverse effect on hands as outcome

Adverse effects on hands	Odds ratio	*SE*	*p*	95% Confidence interval	
Sex					
Female	1.56	0.24	0.00	1.16	2.11
Male	1.00	(base)			
Age					
21–30	3.14	1.05	0.00	1.63	6.06
31–40	1.84	0.60	0.06	0.97	3.49
41–50	1.70	0.55	0.10	0.91	3.20
51–60	1.70	0.55	0.10	0.90	3.21
61+	1.00	(base)			
Time of use per day					
<1 h	1.00	(base)			
1 < h < 3	2.39	0.54	0.00	1.53	3.74
3 < h < 6	3.37	0.73	0.00	2.21	5.15
>6 h	4.37	1.01	0.00	2.78	6.87
Job	1.09	0.09	0.28	0.93	1.27
Sector	0.96	0.03	0.25	0.90	1.03

The regression analysis showed an OR of 1.56 for women, an higher OR for young adults (the highest risk has been observed between 21 and 30 years) and an increased risk with time of utilization of the gloves; the job and the working sector did not have some role. The model of regression had *p <* 0.001, R2 of 0.06, sensitivity of 71.07% and specificity of 53.55%; the model describes well the set of observations (goodness of fit *p =* 0.41).

Concerning the adverse effects linked to bonnet use, we found a statistically significant (*p <* 0.05) correlation between adverse effects and dermatological pathologies, age, sex, and time of utilization; there was no correlation with age (*p =* 0.12) and type of PPE (*p =* 0.065).

The regression analysis showed an OR of 1.87 for women, an higher OR for young adults (the highest risk has been observed between 21 and 30 years) and an increased risk with time of utilization of the gloves; the job and the working sector did not have some role (Table 6). The model of regression had *p <* 0.001, R2 of 0.04 and specificity 100%, of 53.55%; the model describes well the set of observations (Goodness of Fit *p =* 0.47).

**TABLE 6:** Results from multiple logistic regression analysis with adverse effect related to bonnet use as outcome

Bonnet‐related adverse effects	Odds ratio	*SE*	*p*	95% Confidence interval	
Sex					
Female	1.87	0.55	0.03	1.05	3.31
Male	1.00	(base)			
Time of use per day					
<1 h	1.00	(base)			
1 < h < 3	0.70	0.35	0.47	0.27	1.84
3 < h < 6	1.13	0.49	0.78	0.48	2.64
>6 h	2.23	0.85	0.04	1.05	4.71
Job	1.26	0.19	0.12	0.95	1.68
Sector	0.98	0.05	0.70	0.88	1.09

Concerning the mask‐related adverse effects, we found a statistically significant (*p <* 0.05) correlation between adverse effects and dermatological pathologies, age, sex, job, working sector and time of utilization; there was no correlation type of PPE (*p =* 0.299).

The regression analysis showed an OR of 2.69 for women, an higher OR for young adults (the highest risk has been observed between 21 and 30 years) and an increased risk with time of utilization of the mask (significant for a >6 h time of use with an OR of 5.68); the job and the working sector did not have some role (Table 7). The model of regression had *p <* 0.001, R2 of 0.07 sensitivity of 43.98% and specificity of 77.57%, %; the model describes well the set of observations (Goodness of Fit *p =* 0.15).

**TABLE 7:** Results from multiple logistic regression analysis with adverse effect related to mask use as outcome

Mask‐related adverse effects	Odds ratio	*SE*	p	95% Confidence interval	
Sex					
Female	2.69	0.42	0.00	1.98	3.65
Male	1.00	(base)			
Time of use per day					
<1 h	1.00	(base)			
1 < h < 3	1.60	1.45	0.61	0.27	9.49
3 < h < 6	3.17	2.54	0.15	0.66	15.28
>6 h	5.68	4.51	0.03	1.20	26.96
Type of mask	1.21	0.10	0.02	1.03	1.41
Job	1.18	0.09	0.03	1.02	1.38
Sector	0.93	0.03	0.03	0.87	0.99

## 4 DISCUSSION

There is growing evidence that COVID‐19 is associated with a variety of skin reactions, often related to a secondary cell‐mediated immune response following the initial viral infection. The most common cutaneous manifestations in adults are generalized or localized maculopapular eruptions, urticaria, pseudochilblain and acro‐ischemic lesions, varicelliform rash, livedoid lesions, erythema multiforme‐like vasculitis, herpes lesions, purpuric lesions, acute generalized exanthematous pustulosis (AGEP)‐like rash. ^5^, ^6^

On the other side, COVID‐19 can affect skin indirectly: protecting healthcare workers (HCWs) requires the use of personal protective equipment (PPE), but occupational dermatitis caused by PPE is an emerging problem in the midst of the COVID‐19 pandemic. PPE recommendations for healthcare professionals usually include a mask, eye protection (goggles or face shield), bonnet, insulated gloves and gowns, and hand hygiene with alcoholic cleaners causing adverse effects on their skin integrity. According to early data from China and other publications, most HCWs experienced xerosis, pruritus, erythema, papules and maceration.^7^ Moreover, this is an emerging occupational health issue: a survey administered to 1223 Italian HCWs highlighted that 90 medical surveillance visits were requested due to PPEs related dermatological issues: 30 cases were recognized limitations in working duties and in one case the worker was deemed not fit to keep working.^8^ Irritant contact dermatitis (ICD), allergic contact dermatitis (ACD), pressure lesions/skin damage due to frequent washing or alcohol‐based disinfectants or prolonged use of PPE have been reported in the literature.^9^ Since the COVID‐19 will likely be a persistent and recurring problem, frontline staff dermatitis rates are likely to increase accordingly and must be considered.^10^

A survey on 1184 health workers with different occupational sectors was conducted throughout the Italian national territory. Adverse skin reactions caused by the use of gloves, masks and bonnets and the possible exacerbation of pre‐existing pathologies were investigated. The observational study by Lin P et al.^11^ and the systematic review by T. Montero‐Vilchez^12^ were concordant with our sample in showing that a previous history of atopy or hand eczema, acne or seborrheic dermatitis and the prolonged use of PPE were significant risk factors. Nevertheless, there is controversial information regarding other kind of risk factors, such as sex.

HCWs suffering from previous inflammatory dermatological conditions such as AD, SD, acne and psoriasis, had an increased risk of experiencing adverse skin reactions to PPE. These dermatological pathologies have a common pathogenic aspect: all of them are characterized by an endogenously impaired or altered epidermal barrier function, microbiome and/or immunology that promote, often in presence of exogenous factors, the development of the characteristic lesions for each skin disease. For this reason, patients with a history of AD, SD, acne and/or psoriasis are more likely to develop inflammatory lesions in correspondence with the use of PPEs compared with the unaffected subjects. It is arguable that beard may be a protective factor on the onset of face and neck dermatitis due to the reduced contact with the mask.

In detail, patients with AD have an impaired epidermal barrier function, even in uninvolved skin. The loss‐of‐function mutations in the structural protein filaggrin is a widely replicated major risk factor for AD and eczemas; this heritable epithelial barrier defect leads to increased penetration of irritants and allergens through the skin followed by polarized Th2 lymphocyte responses with resultant chronic inflammation. Patients with a history of AD or with filaggrin mutations are more likely to develop both allergic and irritant dermatitis; this can explain why there is an increased incidence of eczematous manifestations in those HCWs using PPEs previously suffering of AD. ^13^

Similar considerations can be applied to SD: subjects with a previous history of SD are more likely to develop erythema, scaling and itching in concomitance with PEEs use. In this case, the microbiome composition differs in patients with SD and individuals without the disease. The microbiome dysbiosis, which is an endogenous characteristic of SD patients, can be exacerbated by mask wearing, that can lead to a proliferation of *Malassezia* spp. and sweating with irritant action and worsening of itching.^14^

Regarding PPEs related acne, which may be considered a subtype of acne mechanica, it occurs more frequently in subjects with a previous history of acne vulgaris. In this case, the endogenous skin characteristic that may explain this increased prevalence is hyperseborrhea, that leads to the development of an acne prone skin. The high temperature of the face covered by the mask induces an increased sebum excretion rate by 10% for each 1°C rise. In those subjects with a baseline overproduction of sebum this promotes the occurrence of acneic lesions especially in concomitance with surgical mask use.^15^

Finally, psoriatic patients are more likely to develop inflammatory lesions compared with the unaffected subjects as a result of mask‐related Koebner phenomenon.^16^

All the PPEs adverse effects have shown to be time‐dependent. The prolonged use of these devices promotes the development of a warm, moist, occlusive environment, a well described risk factor for the occurrence of various inflammatory lesions.

Last, our study has put in evidence that women more likely than men report the occurrence of skin problems caused by all the examined PPEs. Possible reasons for these gender disparity can be individuated in the use of cosmetics, that can be a triggering factor for many dermatologic conditions, and in the long‐lasting use of PPEs. To note, some studies have shown that males are observed wearing mask less than females: as the side‐effects of wearing PPEs are time dependent, this can contribute to explain this difference.^17^

Summing up, the main risk factors individuated in this study for the development of PPEs related cutaneous adverse effects are previous dermatological diseases, time of utilization and female sex. However, these results may have some limitations. In particular, it is possible that symptomatic staff is more inclined to complete the survey, as far as those subjects who had experienced yet cutaneous pathologies, leading to a selection bias. Moreover, the significant gender disparity of PEEs related manifestation can carry a response bias: some studies indicate men and women have differing perceptions of face masks; women were more likely to perceive face masks as being uncomfortable, that may lead to a misrepresentation of the reported cutaneous symptoms.^18^ Further studies are needed to evaluate whether and how vaccines have changed the habits of healthcare personnel in wearing masks.

## 5 CONCLUSIONS

Although data from Europe are limited, our study highlighted the importance of the problem of PPE skin reactions in a large sample of Italian healthcare professionals.

Most healthcare professionals currently have been vaccinated; however, the use of PPE has not changed and skin adverse reactions still represent a global problem with no end date in sight. National data for affected healthcare professionals could contribute to a better understanding of the problem and prevention initiatives in the workplace are desirable.

## CONFLICT OF INTEREST

The authors declare no conflicts of interest.

## AUTHORS CONTRIBUTION

Conceptualization, design of the study: Ilaria Proietti, Ivan Borrelli. Acquisition of data, writing‐original draft: Nevena Skroza, Paolo Emilio Santoro, Maria Rosaria Gualano, Nicoletta Bernardini, Alessandra Mambrin, Ersilia Tolino. Writing‐review and editing: Anna Marchesiello, Federica Marraffa, Simone Michelini, Giovanni Rossi, and Salvatore Volpe. Supervision: Walter Ricciardi, Umberto Moscato, Maria Rosaria Gualano, and Concetta Potenza.

## ETHICS STATEMENT

All procedures performed in studies involving human participants were in accordance with the ethical standards of the institutional and/or national research committee and with the 1964 Declaration of Helsinki and its later amendments or comparable ethical standards. All patients provided written informed consent prior to any study‐related procedures.

## Data Availability

The data that support the findings of this study are available from the corresponding author upon reasonable request.
